# Short-term effects of an educational intervention on physical restraint use: a cluster randomized trial

**DOI:** 10.1186/1471-2318-6-17

**Published:** 2006-10-26

**Authors:** Anna R Huizing, Jan PH Hamers, Math JM Gulpers, Martijn PF Berger

**Affiliations:** 1 Department of Health Care Studies, Section of Nursing Science, Maastricht University, P.O. Box 616, 6200 MD Maastricht, The Netherlands; 2 Verpleeghuis Lückerheide (Nursing Home), MeanderGroep Zuid-Limburg, St. Pieterstraat 145, 6463 CS Kerkrade, The Netherlands; 3 Department of Methodology and Statistics, Maastricht University, P.O. Box 616, 6200 MD Maastricht, The Netherlands

## Abstract

**Background:**

Physical restraints are still frequently used in nursing home residents despite growing evidence for the ineffectiveness and negative consequences of these methods. Therefore, reduction in the use of physical restraints in psycho-geriatric nursing home residents is very important. The aim of this study was to investigate the short-term effects of an educational intervention on the use of physical restraints in psycho-geriatric nursing home residents.

**Methods:**

A cluster randomized trial was applied to 5 psycho-geriatric nursing home wards (*n *= 167 residents with dementia). The wards were assigned at random to either educational intervention (3 wards) or control status (2 wards). The restraint status was observed and residents' characteristics, such as cognitive status, were determined by using the Minimum Data Set (MDS) at baseline and 1 month after intervention.

**Results:**

Restraint use did not change significantly over time in the experimental group (55%–56%), compared to a significant increased use (*P *< 0.05) in the control group (56%–70%). The mean restraint intensity and mean multiple restraint use in residents increased in the control group but no changes were shown in the experimental group. Logistic regression analysis showed that residents in the control group were more likely to experience increased restraint use than residents in the experimental group.

**Conclusion:**

An educational programme for nurses combined with consultation with a nurse specialist did not decrease the use of physical restraints in psycho-geriatric nursing home residents in the short term. However, the residents in the control group experienced more restraint use during the study period compared to the residents in the experimental group. Whether the intervention will reduce restraint use in the long term could not be inferred from these results. Further research is necessary to gain insight into the long-term effects of this educational intervention.

## Background

The use of physical restraints in psycho-geriatric nursing home residents is a common procedure in Dutch nursing homes. The prevalences reported in the literature range from 49 to 61 percent [1-3]. International prevalence values range from 15% to 66% in nursing homes [4]. A physical restraint is defined as *any limitation on an individual's freedom of movement by using such devices as a 'geriatric' chair with table, belts tied to a chair or bed and bed rails *[5]. Although the use of various types of restraint have been reported in the literature, some studies do not categorize the use of bed rails as a restraint method [6]. Physical restraint use seems to occur most often in nursing home residents with poor mobility, high dependency and impaired cognitive status [2,7-9]. Other characteristics, such as old age and fall risk, have also been related to the use of physical restraints [4]. The main reason for using physical restraints is the prevention of falls and fall-related injuries [2,5,7-10]. Hamers *et al*. (2004) reported that 80% of the restrained residents in Dutch nursing homes were in restraint to prevent falls and fall-related injuries. However, recent studies have shown that restraints are inadequate measures for preventing falls and fall-related injuries [7,10-12]. Capezuti *et al*. (1996) showed that the use of restraints was not associated with a lower risk of falls or injuries in residents likely to be restrained. Furthermore, several studies showed that a decrease in restraint use did not result in an increase in fall incidence and serious fall-related injuries [6,10,12]. Both prolonged and short periods of restraint use have negative physical, psychological and social consequences for nursing home residents [2,4,13-15]. The use of restraints may cause immobility, incontinence, pressure ulcers, depression, agitation, aggression and mortality in residents [15]. Reducing the use of restraints in daily practice is recommended because physical restraints seem to be inadequate and harmful to nursing home residents [7]. A study demonstrated that an educational programme combined with consultation reduces the use of restraints in nursing homes effectively and safely [6]. The effects of these types of intervention on restraint use with Dutch psycho-geriatric nursing homes residents are not yet known. Therefore, it is relevant to gain insight into the effects of these types of interventions on physical restraint use in Dutch nursing homes.

The objective of this study was to investigate whether an educational intervention has an effect on the use of physical restraints in psycho-geriatric nursing home residents. The hypothesis was put forward that an educational intervention will lead to a reduction of restraint prevalence and intensity, to a reduction in multiple restraint use, and to the use of less restrictive restraint types in residents (e.g. the use of infrared systems instead of waist belts). The following research questions were formulated:

1. What is the effect of the educational intervention on restraint prevalence and intensity of use in psycho-geriatric nursing home residents?

2. What is the effect of the educational intervention on multiple restraint use in psycho-geriatric nursing home residents?

3. What is the effect of the educational intervention on different restraint types used in psycho-geriatric nursing home residents?

## Methods

### Design and sample

A cluster randomized trial was applied to five psycho-geriatric nursing home wards that belonged to one nursing home. The total number of subjects was 167 residents with dementia. Residents suffering from Korsakov's disease and psychiatric diseases were excluded because these residents in general differ from other residents with dementia (e.g., in being younger and having better mobility) and live in special Korsakov's or psychiatric wards in the nursing homes. The wards were assigned at random to either educational intervention (three experimental wards) or control status (two wards). Nurses in the experimental group attended an educational programme on restraint use. Furthermore, consultation with a registered nurse specialized in the use of restraints and in their reduction (nurse specialist) was introduced on the experimental wards. There was no educational intervention in the control group and residents received the normal care.

### Intervention

The intervention consisted of an educational programme combined with consultation with a nurse specialist [6]. The educational programme developed was based on an educational programme of restraint use in Dutch hospitals [16] and on advice of the Dutch Institute for Healthcare Improvement (CBO) about the decision-making process concerning restraint use in care situations [17]. The educational programme was designed to encourage nurses to embrace a philosophy of restraint-free care and be familiar with techniques of individualized care [18]. The educational programme was taught by the nurse specialist and was carried out over a two-month period. Several subjects concerning physical restraints were discussed during five meetings each lasting for two hours, such as the decision-making process towards restraint use, the effects and consequences of restraint use, strategies to analyse risk behaviour of residents and alternatives for restraints. Nurses were also invited to discuss real-life cases during the educational meetings. The nurses could, therefore, combine practical experience with information from the educational programme. There are indications in the literature that interactive and personal educational meetings are more effective than passive education [19]. Therefore, this educational programme consisted of small-scale meetings with an active learning environment for the nurses. The basic principle for selection of nurses for the educational programme was the inclusion of 'key figures' [19] and the inclusion of nurses with different degrees of innovativeness (different types of 'adopters') [20]. Seven nurses, about one third of the nurses per ward and including the charge nurse, from each experimental ward, were invited to attend the meetings. A total of 23 nurses were divided into three groups. Each group consisted of nurses from different wards and 1 charge nurse, in order to promote the exchange of knowledge and experiences between wards. A plenary session, lasting for one and a half hours, was organized after the five educational meetings for all the nurses of the experimental wards to inform them about restraint use and restraint-free care.

The consultation with the nurse specialist focused on supporting nurses in achieving restraint-free care and complying with the decision-making process concerning restraint use as defined in the Dutch guideline for restraint use in care situations [17,18]. The nurse specialist was, therefore, available for consultation for 28 hours a week, visited the wards once a week, attended multidisciplinary meetings about residents and stimulated nurses to use alternatives for physical restraints, such as electronic devices. During the visits to the wards and the multidisciplinary meetings the nurse specialist evaluated the use of restraints in residents and discussed difficulties in achieving restraint-free care.

### Data collection

Data was collected via observers and from questionnaires at baseline (November 2003) and 1 month post-intervention (June 2004). Restraint use with psycho-geriatric nursing home residents was measured during observations. Restraint use was confirmed visually by independent, trained observers on four separate occasions during a 24-hour period for each measurement. The observers (two nurses, one occupational therapist and one member of management) were not told to the exact design of the study, the intervention and the division into experimental and control wards. All three shifts were included in the observations and the day of visit to each unit was randomized to discourage any artificial removal of restraints by staff [6]. The restraint prevalence, intensity, types and multiple restraint use were determined. Restraint prevalence was defined as the percentage of residents observed restrained at any time during the 24-hour period. Restraint intensity indicated the number of times in four observations that a particular resident was restrained. Restraint types were also recorded in order to gain insight into the types of restraint used with residents. Any device with limitation on an individual's freedom of movement was regarded as a restraint. Examples of restraint types are chairs with tables, belts tied to a chair or bed, bilateral bed rails, sleep suits, special sheets (a fitted sheet including a coat that encloses a mattress), chairs with a board (a chair with chair legs fixed to a board), infrared systems, safe seats, and deep or overturned chairs. Multiple restraints indicated the number of different restraint types used per resident recorded during the four observations. The value of Cohen's kappa was calculated to test the inter-rater reliability between observers. The value obtained showed that this was good.

The Minimum Data Set (MDS) version 2.1, which is part of the Resident Assessment Instrument (RAI), was used to collect background data, such as age, gender and other characteristics of residents. The MDS was completed by, especially for this study, trained nurses who worked on the wards. The nurses completed the questionnaires for residents that they cared for. Different scales based on items in the MDS were used to determine characteristics of residents. The Cognitive Performance Scale (CPS) (Morris *et al*. 1994) was used to determine the cognitive status of residents. This scale consists of five MDS items. The scores range from 0 (intact) to 6 (very severe impairment). The CPS scale corresponded closely with scores generated by the Mini-Mental State Examination and the Test for Severe Impairment, nursing judgement of disorientation, and neurological diagnosis of Alzheimer's disease and other dementias [21]. Self performance in activities in daily living (ADL) was measured with the MDS ADL Self-performance Hierarchy. This scale is based on four MDS items and the scores range from 0 (independent) to 6 (total dependency) [22]. The Depression Rating Scale (DRS) [23] was used to determine depression in residents. This scale contains seven items from the MDS with scores ranging from 0 to 14. Residents who score ≥ 3 (cut-off score) on the scale need further evaluation to diagnose depression. The Social Engagement Scale (SES) was used to determine social engagement in residents [24]. The scale contains six MDS items with scores ranging from 0 (lowest level of social engagement) to 6 (highest level of social engagement). A mobility scale was developed from seven MDS items to determine mobility in residents. The MDS items were: 1) movement in bed; 2) transfer in and out of bed; 3) transfer to standing up; 4) walking in the room; 5) walking in the corridor; 6) movement in the ward; 7) movement outside the ward. The scores of the mobility scale range from 0 (independent) to 28 (totally dependent). The internal consistency of the scale was high, with the value of Cronbach's alpha = 0.97. Psycho-active drug use was determined from one item (O6) of the MDS and was recorded as yes, no, when necessary, yes and when necessary. The reliability and validity of the Minimum Data Set and related scales were tested in different studies and found to be sufficient [21-26].

The accident registration form was used to determine fall incidence and fall-related injuries [27]. The accident registration form was completed by employees of the wards who witnessed an accident or cared for the resident after the accident, or were informed about the accident by the resident, family or a visitor. Fall incidence was defined as the number of residents with at least one fall during the period of measurement (one month). Fall-related injuries were defined as the number of residents with any injury, from pain to fractures, as a result of a fall incident during the period of measurement.

### Ethical considerations

Approval was obtained from the Medical Ethical Committee of the University Hospital Maastricht and Maastricht University. Representatives of the residents received written information about the study from the nursing home and Maastricht University. Based on this information the representatives were asked for written consent for the use of personal data of the residents in this study. Nurses and other employees of the nursing homes were informed about the study by presentations and written information.

### Data analysis

Descriptive statistics were computed for the characteristics of the residents. The prevalence, intensity and types of restraint use were examined using frequency tables. Means were computed for intensity and the number of different restraint types used per resident. A chi-square test and a t-test were used to investigate whether there was a significant difference between restraint use in the control and experimental group. Fisher's exact test values were computed when a table had a cell with an expected frequency of less than 5. The value of alpha with regard to analyses of restraint types was set at 0.01 in order to correct for multiple testing because of the increasing risk of type 1 errors. McNemar tests and gain scores were calculated and tested by a t-test to investigate changes over time in both groups. Logistic regression analysis was used to compare restraint use post-intervention, controlling for characteristics of residents. Since the number of wards is limited and the variables were not defined on ward level, multilevel analysis was not appropriate. The dependent variable restraint use indicates residents observed under restraint at any time during the 24-hour period. Characteristics of residents that were assumed to be correlated with restraint use, based on literature and baseline differences across the control and experimental groups, were entered as covariates in the logistics regression analysis. These included age, gender, cognitive status, self performance in activities of daily living, depression, social engagement, mobility, fall incidence, fall-related injuries and psychoactive drug use. The variable of psychoactive drug use was dichotomised. Interactions between covariates were tested with chi-square tests (*P *< 0.001). The final logistic model was selected by the backwards stepwise procedure, with a significant level of α = 0.10 for backward deletion.

## Results

### Sample

A total of 167 residents were selected for participation in this study and informed consent was obtained for 157 of them. Unfortunately, 12 residents died before the baseline measurement started. A total of 145 psycho-geriatric nursing home residents were measured at baseline. As can be seen from Table 1 there were no differences between the experimental and control groups in the characteristics of the residents at baseline, except for depression. However, the depression scores in both groups were below the cut-off point, indicating no symptoms of depression. After the baseline measurement, 19 residents (8 in the control group, 11 in the experimental group) dropped out (mortality), while 18 new residents (4 in the control group, 14 in the experimental group) were included. Post-intervention, the control and experimental groups only differed on depression and cognitive status (Table 1).

**Table 1:** Characteristics of psycho-geriatric nursing home residents at baseline (*n *= 145) and post-intervention (*n *= 144)

	**Baseline**			**Post-intervention**		
***Residents' characteristics***	***Missing values***	***Experimental*** ***(n = 83)***	***Control*** ***(n = 62)***	***Missing values***	***Experimental*** ***(n = 86)***	***Control*** ***(n = 58)***

Age in years (mean, SD)	0	82.4 (7.6)	82.3 (6.4)	0	81.8 (7.7)	82.7 (6.6)
Gender	0			0		
Male		18 (21.7%)	18 (29%)		23 (26.7%)	18 (31%)
Female		65 (78.3%)	44 (71%)		63 (73.3%)	40 (69%)
Cognitive status (mean, SD)^1^	1	4.2 (1.7)	4.1 (1.8)	2	4.4 (1.5)*	3.3 (2.0)*
Self performance in activities of daily living^2^	15	3.6 (1.8)	3.5 (1.9)	13	3.7 (1.8)	3.5 (2.0)
Depression^3^	0	2.5 (2.5)*	1.3 (1.8)*	6	2.6 (2.5)*	0.7 (1.3)*
Social engagement^4^	0	1.9 (1.9)	1.3 (1.7)	1	1.4 (1.6)	1.0 (1.5)
Mobility^5^	3	12.0 (11.9)	12.8 (12.0)	4	12.2 (12.2)	13.2 (11.8)
Fall^6^						
Incidence		10 (12%)	6 (9.7%)		4 (4.7%)	7 (12.1%)
Related injuries		9 (10.8%)	2 (3.2%)		2 (2.3%)	2 (3.4%)
Psychoactive drug use^7^	0			0		
No		49 (59%)	28 (45%)		36 (42%)	25 (43%)
Yes		32 (39%)	32 (52%)		45 (53%)	33 (57%)
When necessary		0 (0%)	0 (0%)		2 (2%)	0 (0%)
Yes and when necessary		2 (2%)	2 (3%)		2 (2%)	0 (0%)

*Indicates statistically significant difference between experimental and control group tested with chi-square test or an independent-samples t-test, *P*-value ≤ 0.05

^1^Cognitive scores from MDS Cognitive performance scale, range 0–6; score 0 = Intact, 1 = Borderline intact, 2 = Mild impairment, 3 = Moderate impairment, 4 = Moderate severe impairment, 5 = Severe impairment, 6 = Very severe impairment

^2^Self-performance level from MDS ADL Self-performance hierarchy, range 0–6; score 0 = Independent, 1 = Supervision, 2 = Limited, 3 = Extensive 1, 4 = Extensive 2, 5 = Dependent, 6 = Total dependency

^3^Depression level from MDS Depression rating scale, range 0–14; scores ≥ 3 indicate symptoms of depressions

^4^Social engagement level from MDS Social engagement scale, range 0–6; score 0 indicates low social engagement, score 6 indicates high social engagement

^5^Mobility level from 7 items of the Minimum Data Set, range 0–28; score 0 indicates independent, score 28 indicates total dependency

^6^Fall incidence and fall-related injuries from items of the Accident Registration Form

^7^Psycho-active drug use from items of the Minimum Data Set

Data from residents measured at baseline (*n *= 145) or post-intervention (*n *= 144) will be used to describe the situation for the measurements separately. Data from residents with complete data (*n *= 126) will be used to compare between measurements.

### Restraint prevalence

Physical restraints were used with 85 (59%) residents at baseline. Restraints were used most often at night (57%) compared to restraint use in the morning (32%), afternoon (32%) and evening (39%). As can been seen in Table 2, the prevalence of restraint use at baseline did not differ between the control and the experimental groups. However, at post-intervention the control group had a higher prevalence of restraint use (69%) compared to the experimental group (52%) and more residents in the control group were restrained at night.

**Table 2:** The number of psycho-geriatric nursing home residents restrained at both measurements (absolute numbers and (%))

	**Experimental group** **(*n *(t0)= 83; *n *(t1)= 86)**	**Control group** **(*n *(t0) = 62;*n *(t1) = 58)**
At baseline (=t0)	47 (56.6%)	38 (61.3%)
Morning	29 (34.9%)	17 (27.4%)
Afternoon	26 (31.3%)	20 (32.3%)
Evening	31 (37.3%)	26 (41.9%)
Night	45 (54.2%)	37 (59.7%)

Post-intervention (=t1)	45 (52.3%)*	40 (69.0%)*
Morning	28 (32.6%)	21 (36.2%)
Afternoon	25 (29.1%)	21 (36.2%)
Evening	24 (27.9%)	23 (39.7%)
Night	45 (52.3%)*	40 (69.0%)*

*Indicates statistically significant difference between experimental and control group tested with chi-square test, *P-*value ≤ 0.05

**Figure 1:**
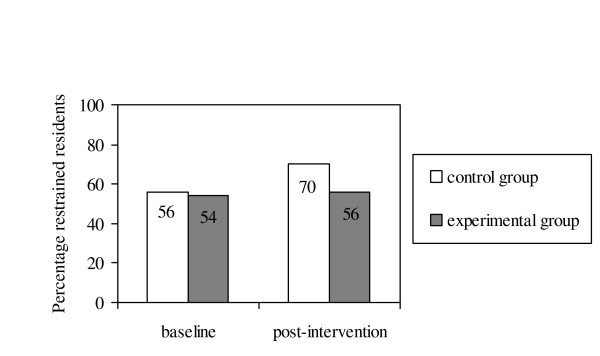
The percentage of psycho-geriatric nursing home residents restrained by group over time (n = 126).

As the study progressed, restraint prevalence in the experimental group did not change significantly. Restraint prevalence during the morning, afternoon, evening and at night did also not change over time. However, restraint prevalence in the control group increased significantly from 56% to 70% (*P *= 0.021) (Figure 1), and there was a statistically significant increase in restraint use in the morning and at night.

### Restraint intensity

The number of times during four observations that a resident was restrained during one day was studied in order to further explore restraint use with nursing home residents. At baseline, residents (*n *= 145) were restrained once (17%), twice (9%), three (6%) or four times (27%) a day, with a mean of 1.59 a day.

Comparison of restraint intensity and the average score of restraint intensity at both measurements showed no statistically significant differences between the experimental and control groups.

As shown in Table 3, there was a significant increase in the mean score of restraint intensity over time in the control group from 1.41 to 1.89. The mean restraint intensity did not change over time in the experimental group. Although the mean score in the control group increased more (gain score = 0.48) compared to the experimental group (gain score = 0.13), the gain score difference was not statistically significant (*P *= 0.133).

**Table 3:** The restraint intensity of psycho-geriatric nursing home residents by group over time (absolute numbers and (%))

	**Experimental group (*n* = 72)**		**Control group (*n* = 54)**	
	
	**At baseline**	**Post-intervention**	**At baseline**	**Post-intervention**
Not restrained	33 (45.8%)	32 (44.4%)	24 (44.4%)	16 (29.6%)
Once restrained	12 (16.7%)	13 (18.1%)	10 (18.5%)	15 (27.8%)
Twice restrained	6 (8.3%)	3 (4.2%)	5 (9.3%)	1 (1.9%)
Three times restrained	4 (5.6%)	2 (2.8%)	4 (7.4%)	3 (5.6%)
Four times restrained	17 (23.6%)	22 (30.6%)	11 (20.4%)	19 (35.2%)

Mean restraint intensity	1.44	1.57	1.41*	1.89*
	(sd = 1.652)	(sd = 1.751)	(sd = 1.596)	(sd = 1.723)

*Indicates statistically significant difference between baseline and post-intervention tested with McNemar test and paired samples t-test (mean scores), P-value ≤ 0.05

### Multiple restraints

The number of different restraint types used per resident during 24 hours was calculated to investigate multiple restraint use. Up to five different types of restraint were used with residents during 24 hours (mean = 1.05, sd = 1.2) at baseline. Most often one restraint type (30%) was used compared to two (16%), three (10%), four (3%) and five (1%) restraint types a day. The control group had a significantly higher mean score of multiple restraint use at post-intervention compared to the experimental group (*P *= 0.033). The change in the average score of multiple restraint between both measurements was calculated for each subject with complete data to further explore changes in multiple restraint use. The mean multiple restraint use in the experimental group did not change over time (*P *= 0.874). However, this did increase in the control group from 1.04 to 1.39 (*P *= 0.006). The differences between groups in gain scores were also statistically significant (*P *= 0.026).

### Restraint types

Based on data from both the measurements, 12 different restraint types were found to be used with nursing home residents. The most frequently used restraints at baseline were bilateral bed rails (57%), sleep suits (14%), belts in bed (11%), belts in chairs (8%), and chairs with a table (8%). Bed rails were the most common restraint type used during the morning, afternoon, evening and at night. Bed rails were used at least once a day with 82 (97%) restrained residents at baseline. Only infrared systems were not used besides or in combination with bilateral bed rails.

Comparison in the use of different restraint types at baseline between the control and experimental groups showed that more sleep suits were used with the control group (23%) compared to the experimental group (7%) (*P *= 0.008). At post-intervention, even more sleep suits were used with the control group (36%) compared to the experimental group (12%) (*P ≤ *0.001).

The use of sleep suits in the control group increased significantly over time from 22% to 38.9% (*P *= 0.012). The increased use of belts in bed with the control group, from 9% to 19%, nearly reached the level of statistical significance (*P *= 0.063). No significant changes occurred over time in the experimental group (Table 4).

**Table 4:** Types of restraints used in residents by group over time (absolute numbers and (%))

	**Experimental group (*n* = 72)**		**Control group (*n* = 54)**	
	
	**At baseline**	**Post-intervention**	**At baseline**	**Post-intervention**
Bilateral bed rail	38 (52.8%)	38 (52.8%)	28 (51.9%)	31 (57.4%)
Sleep suit	6 (8.3%)	8 (11.1%)	12 (22.2%)*	21 (38.9%)*
Belt in bed	10 (13.9%)	7 (9.7%)	5 (9.3%)	10 (18.5%)
Belt in chair	6 (8.3%)	5 (6.9%)	5 (9.3%)	8 (14.8%)
Chair with a table	5 (6.9%)	5 (6.9%)	3 (5.6%)	3 (5.6%)
Chair with a board	4 (5.6%)	2 (2.8%)	0 (0%)	0 (0%)
Special sheet	2 (2.8%)	5 (6.9%)	1 (1.9%)	1 (1.9%)
Deep or overturned chair	0 (0%)	0 (0%)	0 (0%)	1 (1.9%)
Infrared system	0 (0%)	0 (0%)	1 (1.9%)	0 (0%)
Safe seat	0 (0%)	1 (1.4%)	0 (0%)	0 (0%)
Vest with belt	0 (0%)	0 (0%)	1 (1.9%)	0 (0%)
Bedroom door locked	0 (0%)	1 (1.4%)	0 (0%)	0 (0%)

*P -value ≤ 0.01 (correction for multiple testing)

### Logistic regression

Logistic regression analyses were performed to compare restraint use between groups at post-intervention, controlling for ten characteristics of residents (Table 1). Table 5 shows the results of the backwards stepwise logistic regression. The final model demonstrated a significant treatment effect (OR = 0.129) on restraint use. Residents in the experimental group had a lower risk of restraint use compared to the control group. Cognitive status (OR = 2.051) and mobility (OR = 1.732) acted as confounding factors in the logistic regression analysis. The model also demonstrated that the effect of mobility on restraint use is lower for ADL-dependent residents compared to ADL-independent residents.

**Table 5:** Odds Ratios (OR) and 95% Confidence Intervals (CI) for restraint use post-intervention

	**β**	***P-*value**	**OR**	**95% CI**
Treatment (1 = experimental)	-2.049	0.005	0.129	0.031–0.541
Cognitive status	0.719	0.004	2.051	1.253–3.359
Mobility	0.549	0.000	1.732	1.285–2.334
ADL*	-0.314	0.258	0.731	0.424–1.258
ADL* × mobility	-0.066	0.012	0.936	0.889–0.986
Constant term	-2.427	0.004	0.088	

*Self performance in activities of daily living

## Discussion

The predominant finding of this study is that an educational programme for nurses combined with a nurse specialist does not decrease the use of physical restraints in psycho-geriatric nursing home residents in the short term. However, the study showed that residents in the control group experienced more physical restraint use during the study period compared to the experimental group. The educational intervention seems to protect psycho-geriatric nursing home residents from an increasing use of physical restraints.

### Methodological considerations

A total of 167 psycho-geriatric nursing home residents were selected for this study. The results must be interpreted with caution because of the relatively small number of residents. The residents were selected from five nursing home wards that belonged to one nursing home, which may limit the generalization of the study results. In addition, randomization on only five nursing homes wards probably increased the risk of type 2 errors occurring [28]. The nursing home wards were assigned at random to the educational intervention. Efforts were made to reduce contamination bias between wards by limiting information for nurses about the study at the start of the study. The nurses and nurse staff were unaware of the aim and design of the study. After randomization the experimental wards were informed about the precise aim and design of the study and were requested to be careful with the information in regard to the control wards. An educational programme for nurses combined with consultation with a nurse specialist was introduced on the experimental nursing home wards. The implementation of nursing consultation in the nursing home wards is time-consuming. Therefore, the short-term effects of the educational intervention can be attributed only to the educational programme. The nurse specialist fulfilled her tasks during the educational programme. However, time was too short for the nurse specialist to perform all her tasks, such as visiting wards once a week and attending multidisciplinary meetings about residents in the nursing home wards. Follow-up studies are necessary to expand consultation with a nurse specialist in practice and to investigate the long-term effects of the educational intervention. Logistic regression analysis was performed to compare restraint use between groups at post-intervention, controlling for ten characteristics of residents. It might be possible that not all possible confounding factors were included in this analysis, because numerous characteristics of residents have been related to the use of physical restraints in the literature [4].

### Results

An educational programme for nurses combined with a nurse specialist did not decrease the use of physical restraints in psycho-geriatric nursing home residents in the short term. There are some possible explanations for these results. First, changes in care generally take place slowly and change processes require a certain amount of time [19]. Furthermore, reduction of restraint use in practice probably involves a 'paradigm shift' [18]. From viewing behaviour as a problem to be controlled with physical restraints, nurses need to view behaviour as a communication of health state change or a need that is not met [18]. Therefore, one month post-intervention might be too short a period to measure an effect on restraint use. Finally, another explanation for the fact that there was no decrease in physical restraint might be that the short-term effects of the educational intervention can be attributed only to the educational programme. Education is often a necessary first step in a process of implementation of innovations, although, Grol *et al*. (2005) also reported that there is still little research evidence to support the effectiveness of education. Additional interventions are probably necessary to change behaviour and to maintain the changes [19]. The findings of Evans *et al*. (1997) also showed that although education is useful, a far greater effect on reducing restraints is achieved when education is combined with consultation. Therefore, studies concerning the long-term effects of educational interventions on restraint use are recommended.

Logistic regression analysis showed that residents in the control group were more likely to experience increased restraint use than residents in the experimental group. Mobility and cognitive status acted as confounding factors in the regression analysis. This can be confirmed by other studies showing that poor mobility and cognitive status are predictors of restraint use [2,7-9]. The analysis also showed that the control group had a significantly increased use of physical restraints over time compared to no significant changes in the experimental group. Residents became older during the study period, so an increased use of physical restraints in the control group was not unexpected. A recent study about restraint reduction also showed an increased use of restraints in the control group over time [29]. The characteristics of the residents in the control group, with the exception of cognitive status and depression, were similar to the characteristics of those in the experimental group at post-intervention. The residents in the experimental group were cognitively more impaired and had more symptoms of depression. In spite of the poorer cognitive status of residents in the experimental group, nurses were able to care for these residents without an increased use of physical restraints. The lower level of depression in the control group might have influenced the increased restraint use, due to a possible higher activity level associated with less depression. Furthermore, differences in organisational characteristics, such as the workload of the nurses and the staffing level, and other characteristics, like the level of fall prevention on the ward and attitudes of nurses, might explain the increased use of restraints in the control group. However, differences in these characteristics between both groups are unknown. Further research is recommended to gain insight into the relationship between these aspects and the use of physical restraints. Since the use of psycho-active drugs might be considered as chemical restraint and is related to the use of physical restraints, this relationship needs also further investigation.

The results regarding mean restraint intensity and mean multiple restraint use were in agreement with the restraint prevalence – the results showed no decrease in the experimental group and an increase in the control group. The same types of restraint were used with both groups, only sleep suits were used more often with residents in the control group. The hypothesis that the educational intervention leads to the use of less restrictive restraint types with residents could not be confirmed by this study.

Bilateral bed rails are the most frequently used restraint in psycho-geriatric nursing home residents. Bed rails were used at least once a day with 97% of the restrained residents. Use of bed rails can be interpreted as standard for restrained residents based on these results. Hamers et al. (2004) reported that some nurses did not categorize the use of bed rails as a restraint method and probably regarded bed rails as a safe and routine intervention to prevent falls. Some nurses probably translate the use of bed rails as safe patient care [2,30]. The efficacy, hazardous nature and restrictiveness of restraints, such as bed rails, are also under discussion in the literature [14,15,31]. The use of bed rails is, for example, not always defined as a restraint [6,7,12]. Viewing bed rails as a restraint that needs to be reduced in practice is recommended when the ineffectiveness and negative consequences of bed rails are taken into account [32]. Further, more insight into the efficacy, hazardous nature and restrictiveness of physical restraints is necessary to gain a better understanding of restraint use with psycho-geriatric nursing home residents. Therefore, insight into opinions and attitude of, for example, residents, family, nurses and other carers is essential to accomplish restraint-free care.

## Conclusion

The conclusion from this study is that an educational programme for nurses seems to protect psycho-geriatric nursing home residents from an increasing use of physical restraints, but that it does not decrease the use of restraints in residents in the short term. Before we can make recommendations regarding the usefulness of the educational intervention in clinical practice, however, further research is needed. In an ongoing study, with an increased sample size, we examine whether a reduction of restraint use can be obtained by expanding the consultation with the nurse specialist following on the educational programme. That study will also gain more insight into the long-term effects of the educational intervention. In addition, it is important to conduct a process evaluation to explain the success or failure of the intervention. Therefore, it is necessary to get more insight into the influence of organisational characteristics, like workload, autonomy and staff-mix, and the attitude of nurses towards restraint use and change. The challenge still remains to develop measures to expel the use of physical restraints from clinical practice.

## Competing interests

The author(s) declare that they have no competing interests.

## Authors' contributions

All authors contributed to the study conception and design. AH participated in the data collection, data analysis and drafting of the manuscript. JH participated in the data collection and data analysis. MG participated in the data collection. MB participated in the data analysis. All authors critically reviewed the manuscript, read and approved the final manuscript.

## Pre-publication history

The pre-publication history for this paper can be accessed here:


